# Artificial Nerve Containing Stem Cells, Vascularity and Scaffold; Review of Our Studies

**DOI:** 10.1007/s12015-022-10467-0

**Published:** 2022-11-05

**Authors:** Ryosuke Kakinoki, Masao Akagi

**Affiliations:** Department of Orthopedic Surgery, Kindai University Hospital, 377-2 Oono-Higashi, Osaka-Sayama, Osaka, 589-8511 Japan

**Keywords:** Axonal regeneration, Tissue engineering, Artificial nerve, Tubulation, Stem cells

## Abstract

To promote nerve regeneration within a conduit (tubulation), we have performed studies using a tube model based on four important concepts for tissue engineering: vascularity, growth factors, cells, and scaffolds. A nerve conduit containing a blood vascular pedicle (vessel-containing tube) accelerated axon regeneration and increased the axon regeneration distance; however, it did not increase the number or diameter of the axons that regenerated within the tube. A vessel-containing tube with bone-marrow-derived mesenchymal stem cell (BMSC) transplantation led to the increase in the number and diameter of regenerated axons. Intratubularly transplanted decellularized allogenic nerve basal lamellae (DABLs) worked as a frame to maintain the fibrin matrix structure containing neurochemical factors and to anchor the transplanted stem cells within the tube. For the clinical application of nerve conduits, they should exhibit capillary permeability, biodegradability, and flexibility. Nerbridge® (Toyobo Co. Ltd., Osaka, Japan) is a commercially available artificial nerve conduit. The outer cylinder is a polyglycolic acid (PGA) fiber mesh and possesses capillary permeability. We used the outer cylinder of Nerbridge as a nerve conduit. A 20-mm sciatic nerve deficit was bridged by the PGA mesh tube containing DABLs and BMSCs, and the resulting nerve regeneration was compared with that obtained through a 20-mm autologous nerve graft. A neve-regeneration rate of about 70%–80% was obtained in 20-mm-long autologous nerve autografts using the new conduits.

**Graphical Abstract:**

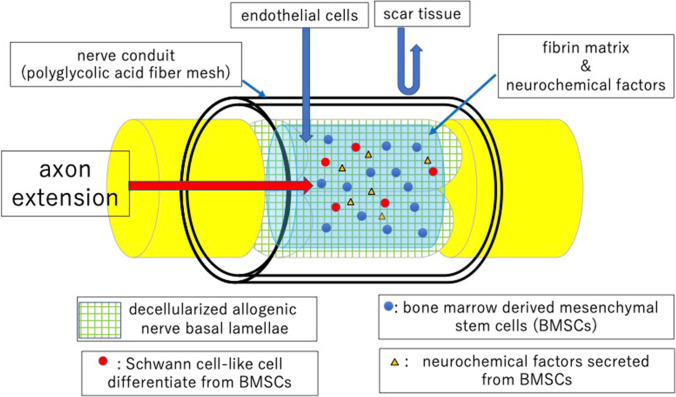

## Introduction

Nerve regeneration through a tube-like material is called tubulation [1, 2]. Williams et al. studied the process of nerve regeneration via tubulation [3]. According to their study, a fibrin matrix containing various neurochemical factors is formed within the tube in the first step of nerve regeneration via tubulation. The next step consists of capillary extension from both nerve stumps into the fibrin matrix. As the capillaries extend, Schwann cells migrate into the fibrin matrix from both nerve stumps. Finally, axons extend into the fibrin matrix from the proximal nerve stump.

However, several issues remain to be addressed before the tubulation technique can be applied in clinical settings. First, there is a limit to the distance across which axons can regenerate via tubulation. Regarding the rat sciatic nerve, the maximum distance of axonal regeneration through a silicone tube was reported to be 10 mm [4]. Second, less-dense populations of axons and those with a smaller diameter regenerate through a conduit compared with autologous nerve graft. The present goal of tubulation is to create a conduit for nerve regeneration that is compatible with that of autologous nerve grafts. We have attempted to promote peripheral nerve regeneration based on four main concepts of techniques of tissue engineering, which include the promotion of intratubular vascularity and the transplantation of cells, scaffolds, and growth factors into the tubes.

### Study 1: Intratubular Vascularity

At the beginning of this research, we attempted to promote nerve regeneration through a silicone tube by adding vascularity into the tube.

**Fig. 1:**
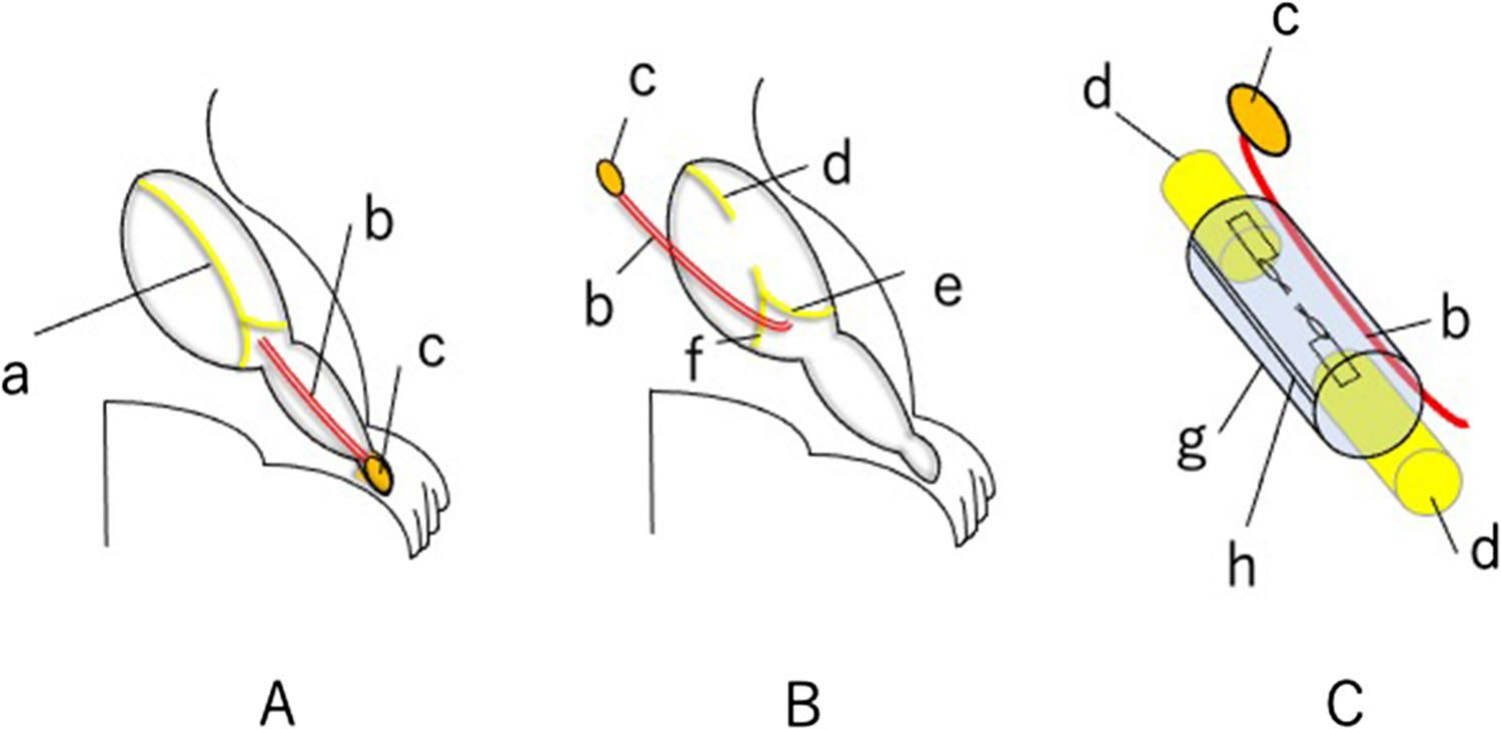
Creation of a vessel-containing tube in a rat hind limb. **A**: A myocutaneous flap supplied by the sural vessels was elevated. **B**: The sural vascular pedicle was separated from the sural nerve and turned proximally. **C**: Through a longitudinal slit in the tube, the sural vascular pedicle was inserted into the tubular lumen. The sciatic nerve stumps were sutured to either end of the tube. **a**, Sural nerve; **b**, sural vascular pedicle; **c**, myocutaneous flap supplied by the sural vessels; **d**, sciatic nerve stump; **e**, tibial nerve; **f**, peroneal nerve; **g**, silicone tube; **h**, longitudinal slit (this was sealed with liquid silicone after vascular insertion)

**Fig. 2:**
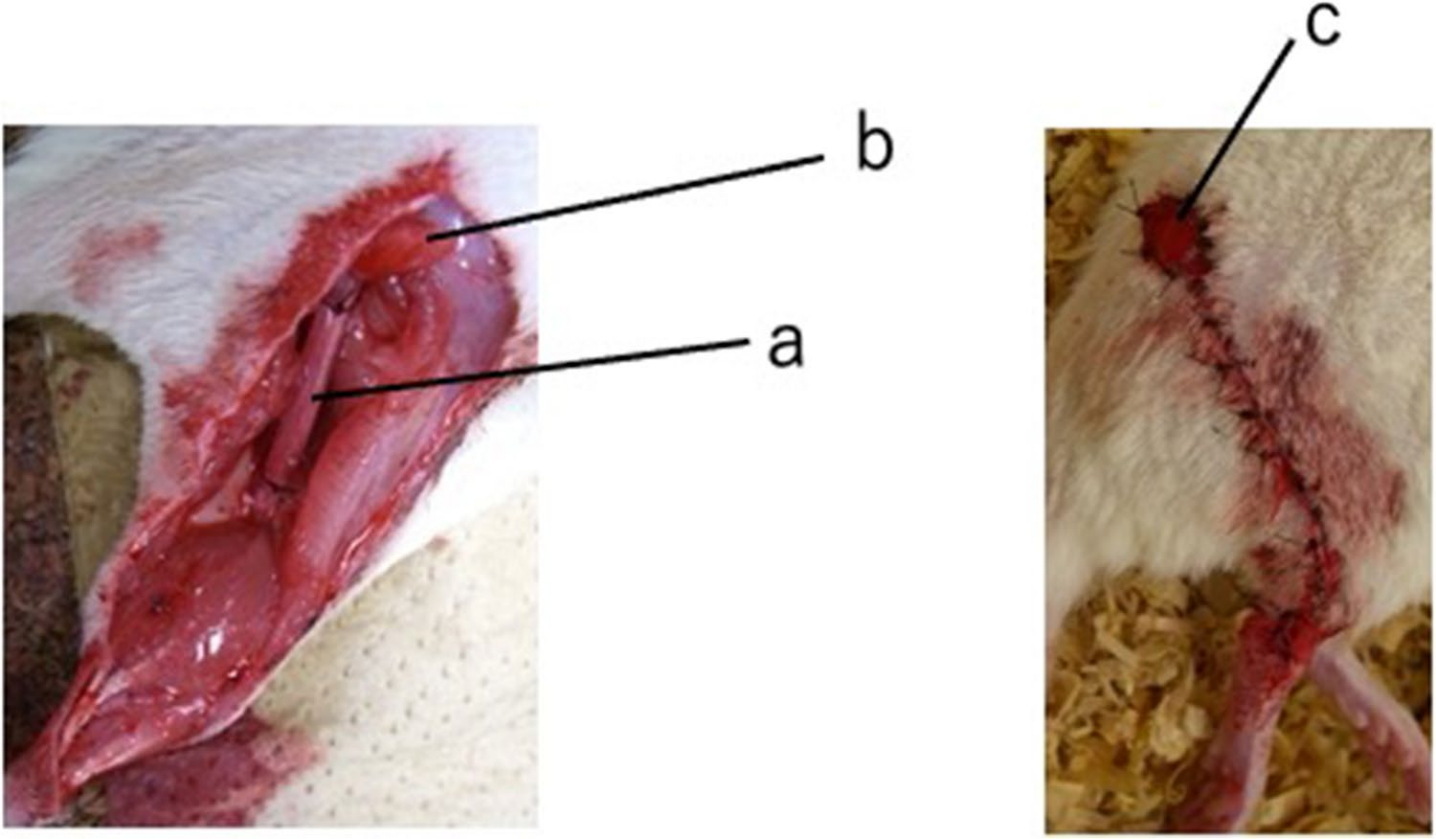
Intraoperative (left) and postoperative (right) images of the vessel-containing tube model in rats. Left: Vessel-containing tube. **a**, vessel-containing silicone tube; **b**, myocutaneous flap. Right: A myocutaneous flap was used as a flap for monitoring sural vessel vascularity

**Fig. 3:**
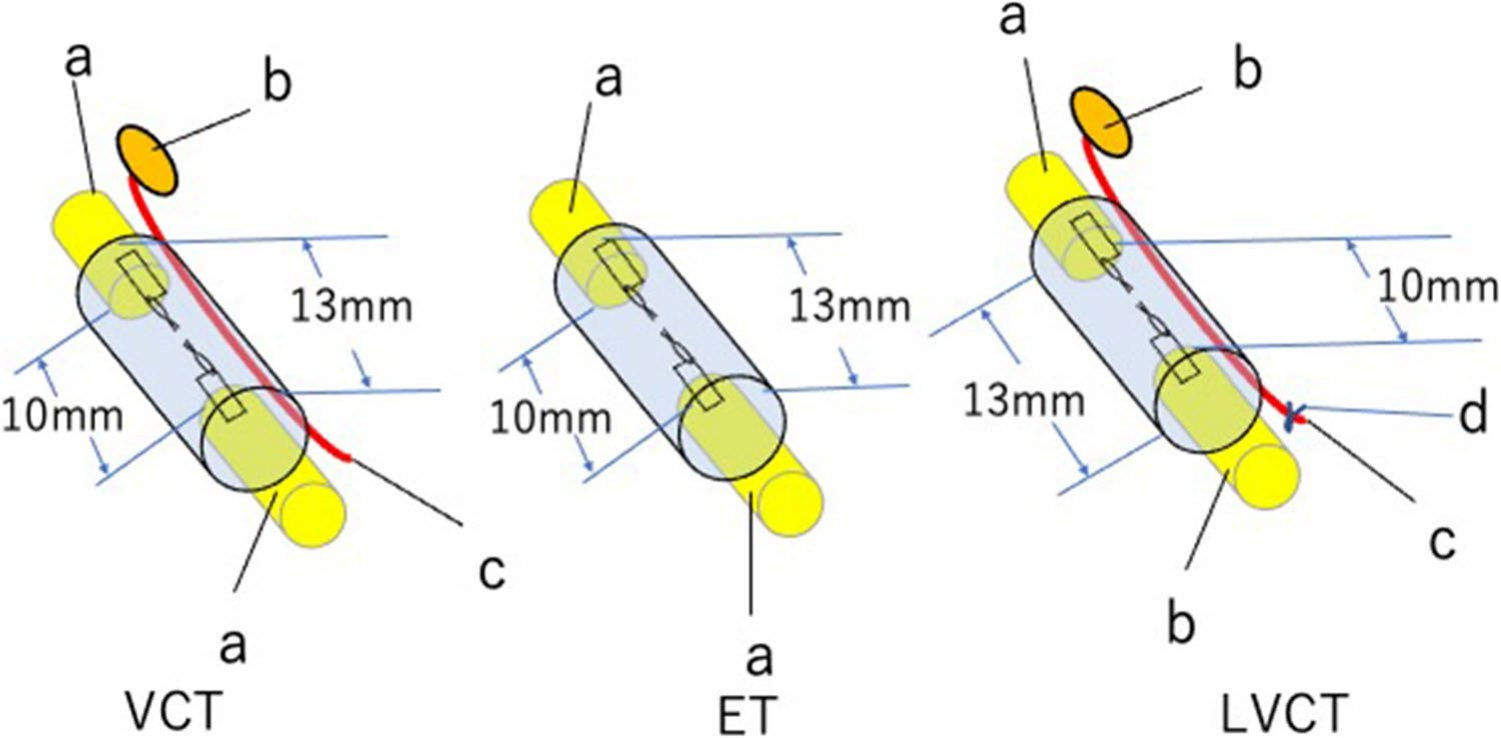
Experimental groups: VCT, ET, and LVCT. VCT, a 13-mm-long sural vessel-containing silicone tube onto each end of which the sciatic nerve stumps were sutured, leaving a 10-mm interstump gap. ET, a 13-mm-long silicone tube without the vascular pedicle bridging the sciatic nerve stumps, leaving a 10-mm interstump gap. LVCT, a VCT tube with the vascular pedicle ligated at the popliteal fossa. **a**, sciatic nerve stump; **b**, myocutaneous flap; **c**, sural vessel pedicle; **d**, ligation of the sural vascular pedicle

We created a vessel-containing tube model in a rat hind limb (Fig. 1) [5]. A sural vascular pedicle was elevated from the ipsilateral lower leg, turned proximally to the thigh, and inserted into a silicone tube (inner diameter 3 mm) through a longitudinal slit in the tube. The sciatic nerve stumps were joined to either end of the silicone tube, leaving a 10-mm interstump gap (Fig. 2). The slit was then sealed with liquid silicone. Three experimental groups were prepared (Fig. 3): VCT group: a 10-mm interstump gap created in a rat sciatic nerve was bridged using a vessel-containing silicone tube; ET group: a 10-mm gap was bridged by an empty silicone tube; and LVCT group: the sural vascular pedicle inserted into a vessel-containing tube with a 10-mm interstump gap was ligated at the origin of the vascular pedicle around the knee.

The angiograms of the empty tube and the vessel-containing tube recorded at 3 weeks after surgery demonstrated that a well-developed capillary network was formed around the inserted vessels in the vessel-containing tube, whereas a few capillaries were observed at the mid portion in the empty tube [5]. The motor nerve conduction velocity (MCV) and compound muscle action potentials in the pedal adductor muscles of the rat hind limbs revealed that the VCT group was significantly superior to the ET and LVCT groups at 6 and 12 weeks after surgery; however, at 24 weeks, there were no significant differences in the two parameters among the three groups. The same tendency was detected in the histomorphometric studies, including the total number of myelinated axons and the myelinated axon diameters measured on the sections harvested from the most distal part of each regenerated nerve [5]. We were able to successfully increase the nerve regeneration distance up to 25 mm through a vessel-containing tube in the rat sciatic nerve [6, 7].

In summary, vessel-containing tubes enhanced the rate of axon regeneration and the distance across which axons were able to regenerate. However, they did not increase the number or the diameter of the regenerated axons.

### Study 2: Intratubular Cell Transplantation

Our next challenge was to increase the number and the diameter of axons that regenerated in the vessel-containing tubes, focusing on cells. There are several options regarding cells that could promote nerve regeneration, including Schwann cells [8], adipose tissue stem cells [9], bone-marrow-derived mesenchymal stem cells (BMSCs) [10, 11], iPS cells [12, 13], gingival mesenchymal stem cells [14], etc. Among them, we chose BMSCs, as they reportedly have a potential to differentiate into various cell lineages, including bone, cartilage, fat, and glial cells [15–18], as well as to secrete various neurochemical [19–22] and neoangiogenetic [23–26] factors. Moreover, the culture and handing of these cells are extremely easy. Our hypothesis for BMSC transplantation into a vessel-containing tube was as follows. BMSCs transplanted into a vessel-containing silicone tube can survive by obtaining oxygen and nutrition from the transplanted vascular pedicle. The BMSCs then differentiate into Schwann cell-like cells in the intratubular environment, which contains neurochemical factors secreted by the nerve stumps and the transplanted BMSCs themselves. Finally, the Schwann cell-like cells promote axon regeneration [27, 28].

**Fig. 4:**
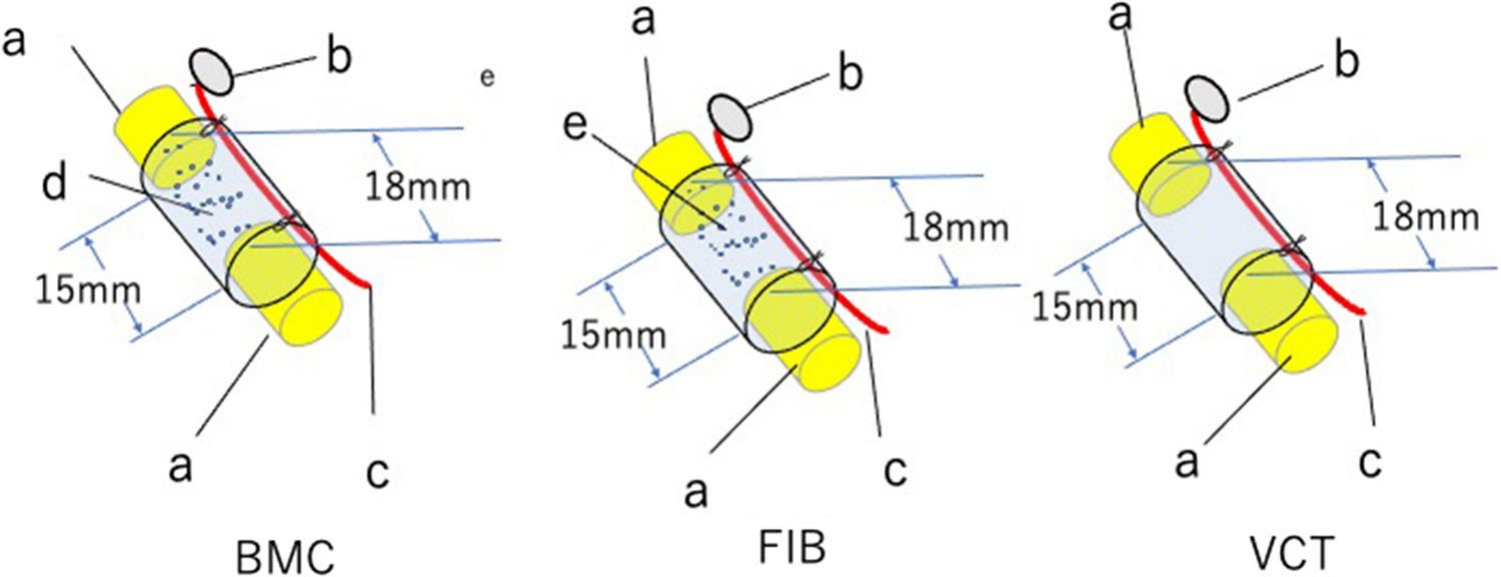
Experimental groups: BMC, FIB, and VCT. BMC, an 18-mm-long sural-vessel-containing silicone tube, at each end of which the sciatic nerve stumps were sutured, leaving a 15-mm interstump gap, followed by the intratubular transplantation of 1 × 10^7^ BMSCs. FIB, an 18-mm-long sural-vessel-containing silicone tube, at each end of which the sciatic nerve stumps were sutured, leaving a 15-mm interstump gap, followed by the intratubular transplantation of 1 × 10^7^ fibroblasts. VCT, an 18-mm-long sural-vessel-containing silicone tube, at each end of which the sciatic nerve stumps were sutured, leaving a 15-mm interstump gap; no cells were transplanted into the tube. **a**, Sciatic nerve stump; **b**, myocutaneous flap; **c**, sural vessel pedicle; **d**, transplanted BMSCs; **e**, transplanted fibroblasts

BMSCs were harvested from the bone marrow of the femurs and tibias of isogenic Lewis rats and cultured in vitro [27]. BMSCs with 4–5 passages were harvested. Three experimental groups were created. In the BMSC group, 1 × 10^7^ BMSCs were transplanted into the tubular lumen of a vessel-containing silicone tube. In the FIB group, 1 × 10^7^ fibroblasts were transplanted intratubularly into a vessel-containing silicone tube. In the VCT group, no cells were transplanted into the vessel-containing silicone tube. A sciatic nerve stump gap was set at 15 mm in this study (Fig. 4). Many myelinated axons were observed on a transverse section of the distal part of the regenerated nerves in the BMSC group at 24 weeks. In contrast, in the FIB group, fewer myelinated axons were detected compared with the BMSC group. Intratubular fibroblast transplantation might have deteriorated axon regeneration within the tube. Electrophysiological and histomorphometric studies demonstrated that nerve regeneration in the BMSC group was significantly superior to that detected in the FIB and VCT groups at 24 weeks after surgery [27].

To determine the proportion of transplanted cells that turned into Schwann cell-like cells within the vessel-containing tube with BMSC transplantation, BMSCs harvested from the male rats were cultured in vitro and 1 × 10^7^ BMSCs were transplanted into a vessel-containing tube created in the hind limbs of female Lewis rats. Transverse sections were harvested from regenerated nerves within the vessel-containing tubes with male-rat-BMSC transplantation. In situ hybridization specific to the sex-determining region of the Y chromosome (Sry) and immunochemical staining specific to the glial fibrillary acid protein (GFAP) was performed on these sections. Images of the in situ hybridization merged with the immunostaining revealed that some GFAP-positive cells showed Sry signals [27]. This indicated that some transplanted BMSCs might have differentiated into Schwann cell-like cells. PCR specific to Sry was performed on genomic DNA extracted from the regenerated nerves within the vessel-containing tubes with male rat-BMSC transplantation. The results of the semi-quantitative PCR studies showed that about 30% of cells in the regenerated nerves originated from the transplanted BMSCs [27].

A vessel-containing tube model was also created using the ulnar nerve and the concomitant ulnar vessels in canine forelimbs [28]. Nerve regeneration was compared between the vessel-containing silicone tubes (with a 30-mm interstump gap) with a 30-mm-long autologous ulnar nerve graft. The nerve autologous graft exhibited a significantly better nerve regeneration than did the vessel-containing tube with BMSC transplantation at 12 weeks; however, at 24 weeks, no significant difference was found between them, although the nerve graft showed a tendency toward the production of a better nerve regeneration than did the vessel-containing tube with BMSC transplantation [28].

In summary, vessel-containing tubes with BMSC transplantation increased the number and diameters of axons regenerated through the tubes and were able to bridge a 20-mm interstump gap in rats, which corresponds to a gap of about 6 cm in primates [29].

### Study 3: Intratubular Transplantation of a Scaffold for Nerve Regeneration

Our next challenge consisted of placing a scaffold intratubularly. There are several candidate scaffolds for nerve regeneration. We targeted decellularized allogenic basal lamellae (DABLs) as an intratubular scaffold for nerve regeneration.

**Fig. 5:**
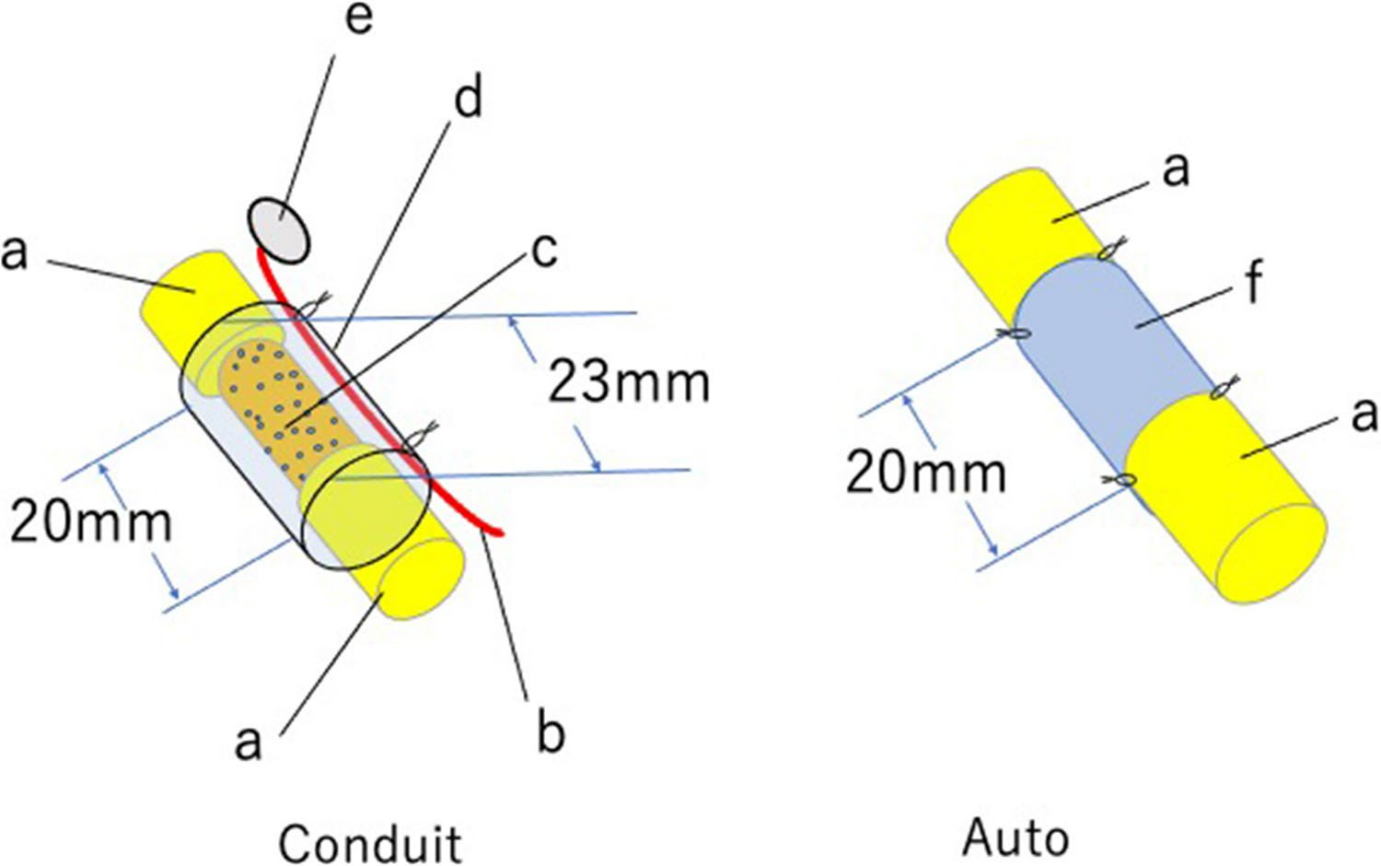
Experimental groups: Conduit and Auto. Left: Conduit group: a 23-mm-long sural vessel-containing silicone tube with DABLs (prepared using the freeze–thaw technique) and 3 × 10^6^ BMSCs intratubularly. Right: Auto group: a 20-mm-long sciatic nerve segment was transected. The segment was reversed and sutured between the proximal and distal sciatic nerve stumps

Sciatic nerve segments were harvested from DA rats (RT-1), which were major histocompatibility mismatched to Lewis rats. DABLs were prepared using the freeze–thaw technique [30]. An immunochemical staining specific to laminin demonstrated the presence of laminin molecules on the basal lamellae of the decellularized nerve [31]. In a Lewis rat, a 20-mm sciatic nerve deficit was bridged with a silicone tube containing a sural vascular pedicle and the 20-mm-long DABLs seeded with 3 × 10^6^ BMSCs. Nerve regeneration in this new tube (Conduit group) was compared with 20-mm-long autologous nerve grafts (Auto group) (Fig. 5). No significant difference was found between the two groups electrophysiologically and histomorphometrically at 24 weeks [32]. BMSCs harvested from GFP-positive Lewis rats were transplanted intratubularly. Regenerated nerves were harvested, and transverse sections were collected 5 mm proximal to the most distal part of each regenerated nerve. This revealed that part of the transplanted BMSCs differentiated into cells with glial cell markers at 6 weeks after transplantation [31, 32].

Immunostaining specific to CD8 was performed on the transverse sections harvested at the mid portion of the fresh allogenic nerve graft (sciatic nerve of DA rats), the regenerated nerve in vessel-containing tubes with transplantation of DABLs (DA rat origin) and BMSCs, and fresh autologous nerve grafts (sciatic nerve of Lewis rats). The antigenicity of DABLs had regressed greatly compared with the fresh allogenic nerves [31, 32]. Anti-laminin staining of DABLs showed the preservation of laminin molecules on the basal lamellae of decellularized allogenic nerves. Because DABLs exhibited minimum antigenicity, preserved laminin molecules, and were not associated with donor-site morbidity, DABLs were considered to be the ideal three-dimensional scaffold for nerve regeneration [31, 32].

### Study 4: Creation of Biodegradable and Capillary Permeable Tubes

In the clinical setting, there are four essential factors for the generation of artificial nerves, as follows. 1. Fibrin matrix formation within the tubular lumen; if a fibrin matrix is not formed within the tubular lumen, axons do not have the space for regeneration. 2. Capillary permeability: if the tube is not capillary permeable, a vascular pedicle needs to be inserted in the tubular lumen. 3. Biodegradability: if the tube is not biodegradable, it needs to be removed after the completion of nerve regeneration. 4. Flexibility: if the tube is hard and inflexible, it cannot be used in the joint parts.

**Fig. 6:**
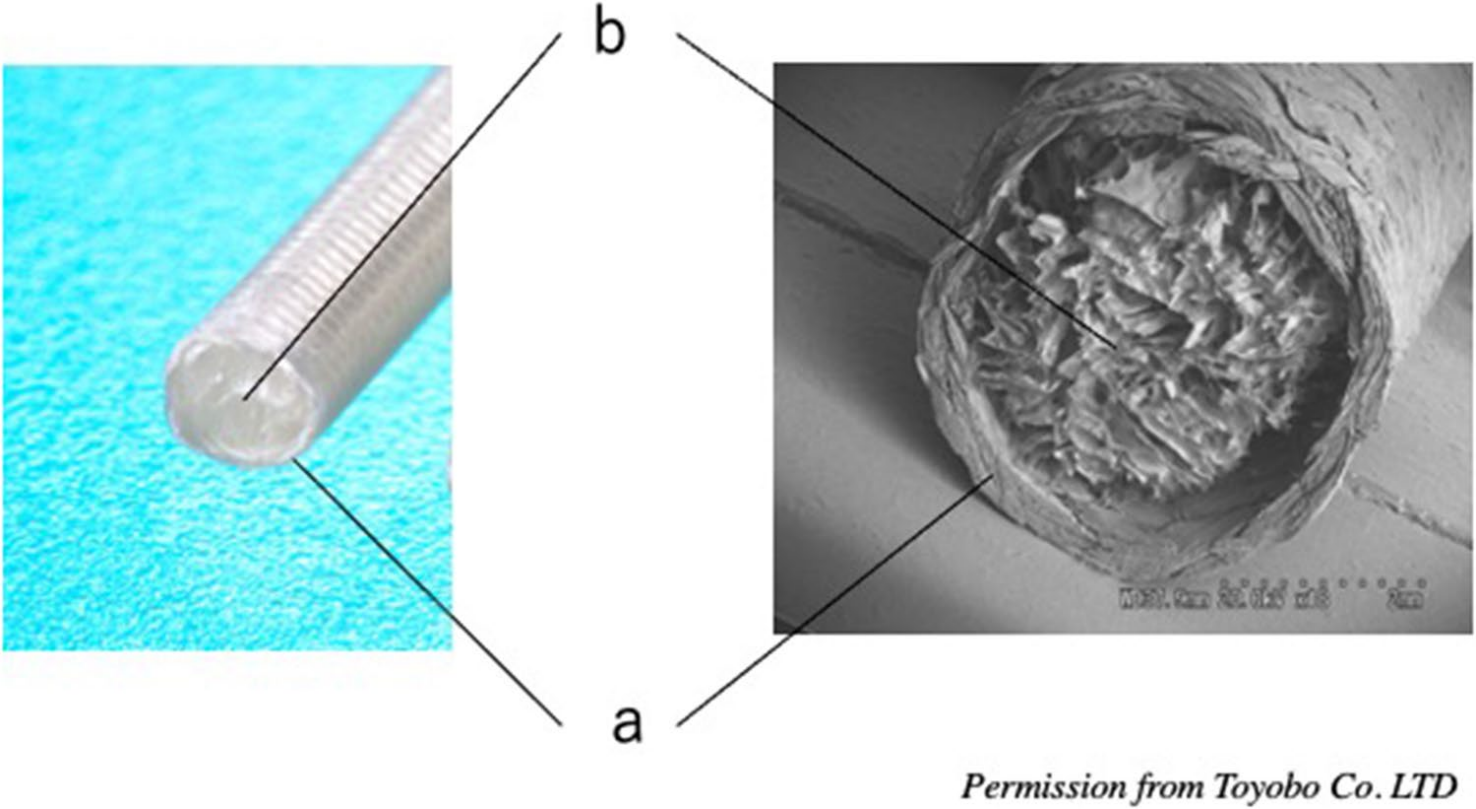
Nerbridge® (*Toyobo, Osaka, Japan*): the outer cylinder is a polyglycolic acid (PGA) fiber mesh, and the inner core is a collagen sponge. Images reproduced with permission from Toyobo Co Ltd

As a nerve conduit, we focused on the outer cylinder of Nerbridge® (Toyobo Co. Ltd., Osaka, Japan). Nerbridge is commercially available as an artificial nerve. The outer cylinder is a polyglycolic acid (PGA) fiber mesh, which allows particles smaller than 600 KD to pass through. The biodegradation starts at 3 months after transplantation in vivo ( https://www.toyobo-global.com/system/files/News_Release/201902/press20180820A.pdf) (Fig. 6).

The use of a capillary permeable tube as a nerve conduit is associated with several risks regarding nerve regeneration within the tube. Although capillaries may enter the tubular lumen from the surrounding tissue, scar tissue may also pass through the tubular wall and enter the tubular lumen. Moreover, neurochemical factors that should accumulate in the tubular lumen may leak out of the lumen, and the fibrin matrix may not be formed within the lumen.

To evaluate the capillary permeability of the outer cylinder of Nerbridge, we created three experimental groups. In the E-tube group, a 5-mm interstump gap in the rat sciatic nerve was bridged by a PGA tube with the sural vessels attached on the outer surface of the tube (extratubularly vascularized tubes). In the I-tube group, a 5-mm interstump gap was bridged by a PGA tube with sural vessels transplanted intratubularly (intratubularly vascularized tubes). In the N-tube group, a 5-mm interstump gap was bridged by a PGA tube without the vascular attachment (non-vascularized tubes). Each tube was wrapped with a 20 × 20 mm silastic sheet to prevent the occurrence of capillary extension at sites other than the nerve stumps joined to either end of the tube and the sural vessels. RECA-1-specific immunostaining was performed on the mid-section of the regenerated nerve of each tube at 6 weeks. No significant difference was found in capillary formation within the regenerated nerves between the E-tube and I-tube groups. In addition, no significant difference was found electrophysiologically and histomorphometrically between the E-tube and the I-tube groups. No neural tissue was formed in the N-tube group [33]. The outer cylinder of Nerbridge allowed capillaries to enter the tubular lumen.

Capillary permeable tubes require a substrate to preserve the fibrin matrix structure intratubularly. We focused on DABLs that were created using chemosurfactants [34], because the DABLs created using the freeze–thaw technique [30] contained cell debris and degenerated myelin, which pose a risk of viral transmission from the donor to the recipient. Electron microscopy examinations revealed that the DABLs created by chemosurfactants exhibited a honeycomb structure containing cell and myelin debris. DABLs created by chemosurfactants may reduce the risk associated with DABL transplantation. We hypothesized that the honeycomb structure of DABLs would help preserve the structure of the fibrin matrix containing neurochemical factors and anchoring transplanted BMSCs within the tubular lumen, leading to good nerve regeneration as well as the reduction of the risk of viral transmission [33].

**Fig. 7:**
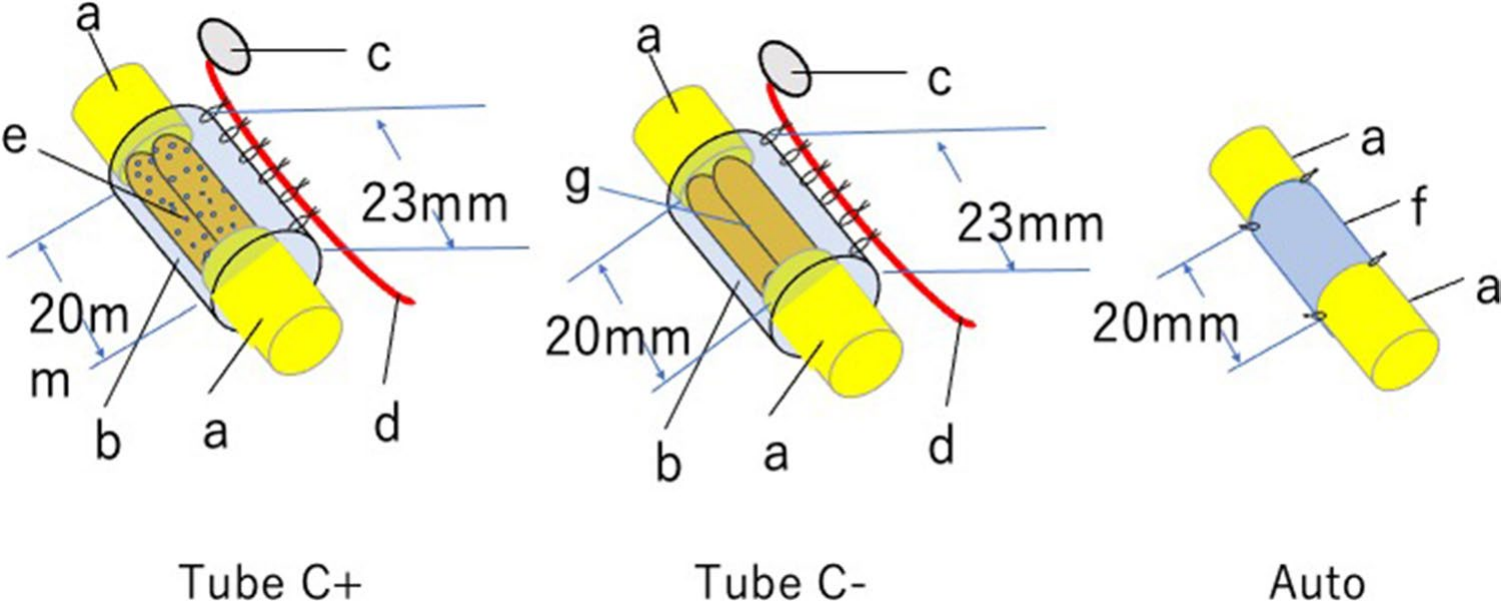
Experimental groups: Tube C + , Tube C–, and Auto. Tube C + : Two 20-mm-long DABLs (harvested from DA rats and prepared using chemosurfactants) seeded with 3 × 10^6^ BMSCs were transplanted in a 23-mm-long polyglycolic acid mesh tube (the outer cylinder of Nerbridge®). A sural vascular pedicle was placed along the conduit. Tube C–: Two 20-mm-long DABLs (harvested from DA rats and prepared using chemosurfactants) without BMSC implantation were transplanted into a 23-mm-long polyglycolic acid mesh tube (the outer cylinder of Nerbridge®). A sural vascular pedicle was placed along the conduit. Auto: A 20-mm-long sciatic nerve segment was transected. The segment was reversed and sutured between the proximal and distal sciatic nerve stumps

**Fig. 8:**
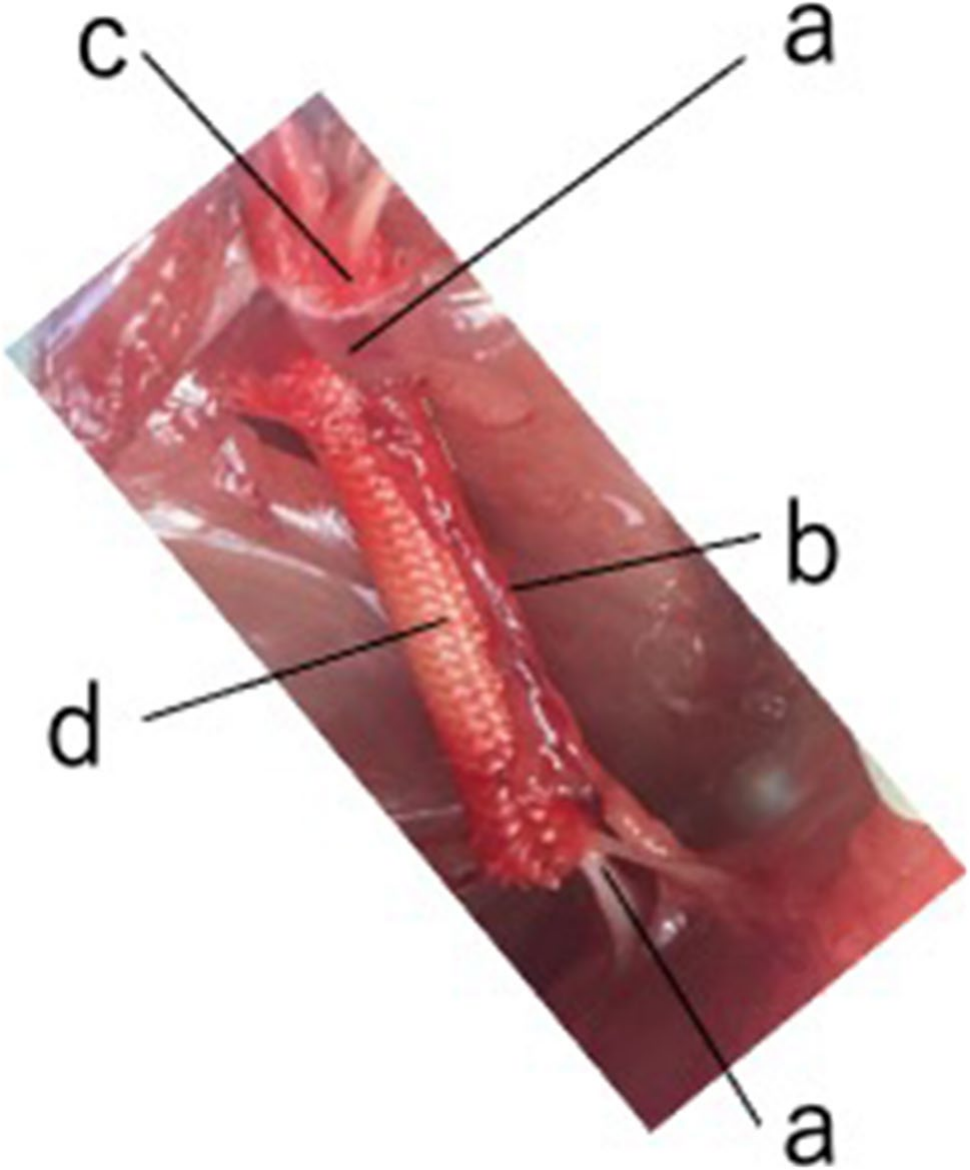
Intraoperative photo of a conduit in the Tube C + group. Tube C + conduit. **a**, sciatic nerve stump; **b**, sural vascular pedicle; **c**, monitor flap; **d**, polyglycolic acid mesh tube

**Fig. 9:**
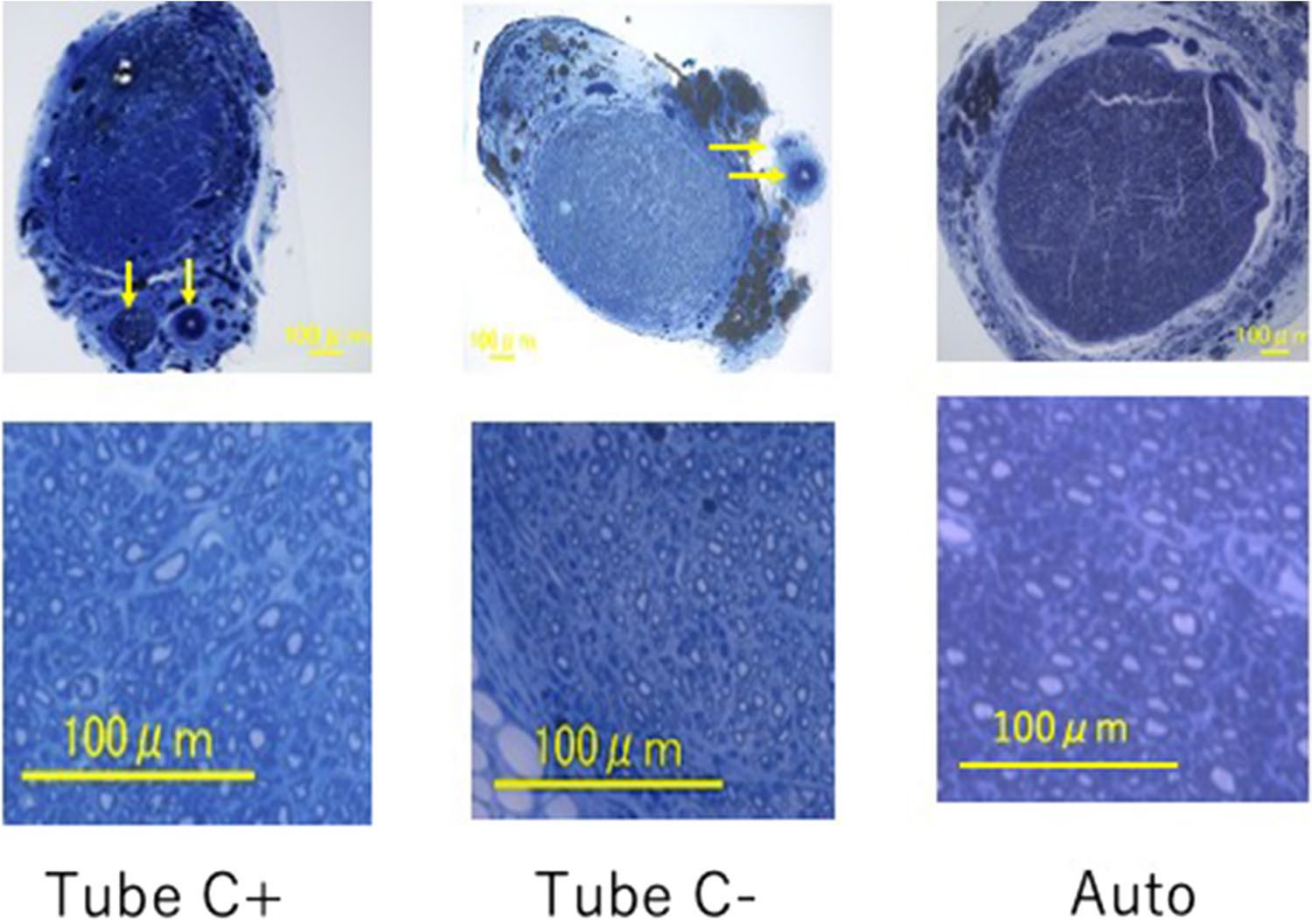
Transverse sections of the most distal part of the regenerated nerves in the Tube C + , Tube C–, and Auto groups. The vascular pedicles are indicated by arrows

We created a nerve conduit using the outer cylinder of Nerbridge (the inner diameter of which was 3 mm) that contained two 20-mm DABL segments seeded with 3 × 10^6^ BMSCs. A sural vascular pedicle was attached to the outer surface of the cylinder. We created three experimental models for comparison of nerve regeneration. In the Tube C + group, a 20-mm interstump gap in the rat sciatic nerve was bridged with an extratubularly vascularized PGA tube containing DABLs and BMSCs. In the Tube C– group, a 20-mm nerve gap was bridged with an extratubularly vascularized PGA tube containing DABLs without BMSC transplantation. In the Auto group, a 20-mm nerve segment was harvested from the sciatic nerve. The segment was inverted and interposed between the sciatic nerve stumps using 10–0-nylon sutures (Figs. 7 and 8). The mean MCV in the Tube C + group was about 92% of that recorded in the Auto group, and the mean CMAP amplitude of the pedal adductor muscle was about 75% that detected in the Auto group. In addition, the mean total myelinated axon number, myelinated axon diameter, and myelin thickness in the Tube C + group were about 70%–80% those recorded in the Auto group (Fig. 9). No apparent scar tissue invasion was observed within the tubular lumen in the Tube C + or Tube C– group. Apparent scar tissue invasion into the tubular lumen was not observed. The outer cylinder of Nerbridge seemed to have a function to prevent scar tissue invasion into the chamber for nerve regeneration [33].

## Discussion

Capillary formation is closely related to axon regeneration. It is known that axon growth cones and capillary tip cells share common signal cues that regulate guidance [34]. Neuropirin and Ephrin receptors are expressed in both the vascular and nervous system [35]. It can be easily anticipated that the acceleration of vascularization within a tube is followed by axon extension. In Study 1, we demonstrated that the nerve regeneration distance and speed were ameliorated by vascular insertion into the tubes; however, the number and the diameter of axons regenerated within the tube cannot be increased solely by promoting vascularity during tubulation [5, 6]. Because several chemical factors are secreted by BMSCs [19–26], intervention using various cells and chemical factors is needed to increase the number and diameter of axons to be regenerated intratubularly.

BMSCs differentiate into several different cell lineages depending on the environment into which they are transplanted. In Study 2, we demonstrated that some BMSCs transplanted into silicone tubes with sural vessel transplantation expressed glia cell markers [18]. However, there are some criticisms regarding the transdifferential capability of BMSCs. Although our previous studies have shown that some transplanted BMSCs exhibited glia cell markers [27, 28, 31, 32] ( https://www.toyobo-global.com/system/files/News_Release/201902/press20180820A.pdf), several authors addressed the possibility of the occurrence of fusion between the implanted cells (transplanted BMSCs) and resident cells (Schwann cells) [36, 37]. It is also unknown whether the transplanted BMSCs exhibiting glia cell markers would really act like Schwann cells in the process of peripheral nerve regeneration. BMSCs have the paracrine ability of producing various neurotropic and neurotrophic factors [19–22], neoangiogenic factors (including angiopoietins, angiopoietin-like factors, and the vascular endothelial growth factor), and the fibroblast growth factor 2 [23–26]. BMSCs may also promote nerve regeneration by producing these endogenous factors.

The fibrin matrix structure is likely to be well preserved in a tube containing an enclosed space in the tubular lumen, whereas the fibrin matrix structure is fragile in the PGA fiber mesh tube because it has substantial interaction between the tubular lumen and the surrounding tissue. Thus, a complement that preserves the structure of the fibrin matrix is needed for nerve regeneration within capillary permeable tubes. The honeycomb structure of the chemically created DABLs might act as a frame for preserving the structure of the fibrin matrix and an anchor to maintain BMSCs within the tubular lumen. Furthermore, laminin molecules remaining on the DABLs might promote axonal regeneration. Collagen fibers or sponge would be a substitute for DABLs from the perspective of a frame structure for fibrin matrix formation. However, the best morphological and material property for fibrin matrix formation remains unclear. Regarding the immunogenicity of DABLs, BMSCs also suppress the immune response by inhibiting T-cell proliferation, which might contribute to the suppression of the immunogenicity of DABLs [38–40].

From study 4, the outer cylinder of Nerbridge seemed to have a potential to allow endothelial cell passage and prohibit scar tissue invasion. Few studies have addressed the relationship between nerve regeneration and the size of molecules that pass through the conduit wall. Aebischer et al. reported that nerve regeneration through a nerve conduit passing molecules smaller than 100 KDa exhibited a significantly improved nerve regeneration compared with a nerve conduit passing molecules smaller than 1000 KDa [41]. It is known that molecules smaller than 600 KDa can pass through the outer cylinder of Nerbridge (https://www.toyobo-global.com/system/files/News_Release/201902/press20180820A.pdf). Further studies are needed in terms of the porous size of the tubular wall. The suitable timing and rate of the degradation of nerve conduits in vivo should also be a topic of future studies.

In conclusion, for clinical applications, nerve conduits should be biodegradable, capillary permeable, and flexible. Vascularity accelerates nerve regeneration and extends the distance across which axons can regenerate. However, to improve the number and the diameter of axons that are regenerated through a conduit, cells, growth factors, and scaffolds are necessary. At present, DABLs seem to be the best scaffold for peripheral nerve regeneration via tubulation from the perspectives of axon extension, laminin preservation, and minimum antigenicity. Further studies are needed to investigate the optimal pore size of the tubular wall, the optimal timing and rate of degradation of nerve conduits, and the optimal number and the most suitable source of stem cells for nerve regeneration via tubulation.

## Data Availability

The authors confirm that the data supporting the findings of this study are available within the articles mentioned in the references.
